# Dynamic Consent: A Possible Solution to Improve Patient Confidence and Trust in How Electronic Patient Records Are Used in Medical Research

**DOI:** 10.2196/medinform.3525

**Published:** 2015-01-13

**Authors:** Hawys Williams, Karen Spencer, Caroline Sanders, David Lund, Edgar A Whitley, Jane Kaye, William G Dixon

**Affiliations:** ^1^Arthritis Research UK Centre for EpidemiologyUniversity of ManchesterManchesterUnited Kingdom; ^2^Centre for Primary CareUniversity of ManchesterManchesterUnited Kingdom; ^3^HW CommunicationsLancasterUnited Kingdom; ^4^Department of ManagementLondon School of Economics and Political ScienceLondonUnited Kingdom; ^5^HeLEX - Centre for Health, Law and Emerging TechnologiesNuffield Department of Population HealthUniversity of OxfordOxfordUnited Kingdom; ^6^Health eResearch CentreUniversity of ManchesterManchesterUnited Kingdom

**Keywords:** dynamic consent, electronic patient record (EPR), medical research, confidentiality, privacy, governance, NHS, data linkage, care.data

## Abstract

With one million people treated every 36 hours, routinely collected UK National Health Service (NHS) health data has huge potential for medical research. Advances in data acquisition from electronic patient records (EPRs) means such data are increasingly digital and can be anonymised for research purposes. NHS England’s care.data initiative recently sought to increase the amount and availability of such data. However, controversy and uncertainty following the care.data public awareness campaign led to a delay in rollout, indicating that the success of EPR data for medical research may be threatened by a loss of patient and public trust. The sharing of sensitive health care data can only be done through maintaining such trust in a constantly evolving ethicolegal and political landscape. We propose that a dynamic consent model, whereby patients can electronically control consent through time and receive information about the uses of their data, provides a transparent, flexible, and user-friendly means to maintain public trust. This could leverage the huge potential of the EPR for medical research and, ultimately, patient and societal benefit.

## The United Kingdom National Health Service

The UK National Health Service (NHS) provides health care for over sixty million citizens throughout their lives. Around one million people are treated every 36 hours [1], with vast amounts of information about patients’ treatment and outcomes collected in their medical records. These “cradle to grave” records are increasingly captured within electronic patient record (EPR) systems rather than on paper. The United Kingdom has national EPR coverage in primary care, and coverage in secondary care (hospital) is increasing. While these records are primarily for health care delivery, such data have huge potential for medical research as well.

The reuse of NHS health care data, such as is routinely stored in these EPRs, has enabled medical research for decades. It has led to a huge expansion in our knowledge, with associated important public health impact, through observational research in areas such as epidemiology, drug safety, outcomes research, vaccines, and health services research. Examples of positive benefit range from how, in the 1940s and 50s, national statistics played a major part in identifying the rising incidence of lung cancer mortality and discovery of its link with smoking, more recently, disproving a suggested link between the measles, mumps, and rubella vaccine and autism [2].

Much research has been possible in England through initiatives such as the Clinical Practice Research Datalink (CPRD), The Health Improvement Network, and QResearch, whereby researchers can access anonymized primary care EPR datasets [3]. Linkage of patient data to national cancer and mortality registers, and to Hospital Episode Statistics, has been available for researchers more recently [4]. As an indication of volume, there are now over 900 peer-reviewed publications from CPRD alone. Linked datasets have also been made available for research in the devolved nations, drawing on the strengths of a unique, widely used Community Health Index number in Scotland [5], and on linkage between health care and social care data in Wales [6].

However, despite clear health care benefits from analysis of high quality data, the success of EPR data for research may be threatened by a loss of trust from patients. Sensitivities abound which need careful management, particularly with respect to the confidentiality of health data. Perception by the public that their personal health care data are being used inappropriately, either shared with organizations such as insurance companies or being sold for profit, leads to distrust. This loss has been exemplified by adverse public reaction to NHS England’s care.data program [7].

## Public Concern and Confidence

Public and patient views about the confidence and trust in the use of EPRs cannot be considered homogeneous. Research has highlighted that the public are often broadly supportive of the use of EPR data for research, while concomitantly having little knowledge of how data held in EPRs are shared, and also articulating concerns about privacy of their data. For example, in a recent study, 80% of UK people supported confidential access to their medical records for research [8]. Nicolson [9] and Kass et al [10] highlight that the public had little knowledge of how their EPR was accessed, used, and shared. Support for EPR data sharing is often grounded in safeguards to protect privacy [8-12]. Concerns expressed within studies [13] and surveys [14] mainly relate to the type of recipient, (ie, anxieties are greater with respect to access by the pharmaceutical industry compared with university academics) anonymity, and types of information shared, with patients less willing to share information as it takes on more of a personal nature [15]. The potential for privacy breaches and data misuse are of particular concern [16]. Privacy invasion concerns were found to be greater among Scottish people, black and minority ethnic groups [12], and among those with lower socioeconomic status or living in rented accommodations [11]. These trends are repeated globally. A recent survey [17] among adult social media users in the United States indicated a willingness to share health data (92% with a medical condition agreed with sharing their health data to help research) despite potential risks (76% worried that health data that they share may be used in detrimental ways).

These concerns about the potential misuse of health data in EPRs are examined in the next section, which focuses on the challenges faced by England’s care.data initiative. The dynamic consent approach, which manages patients’ consent preferences, is presented as a possible solution.

## Concerns About Care.data

Much important UK population health research has successfully used anonymized primary care data. Although much progress has been made, the United Kingdom does not yet have national coverage of EPRs within secondary care. Research into medical conditions managed in hospitals has required bespoke research studies at significant cost and effort, for example, the establishment of national drug safety registers for medication prescribed only by hospital specialists [18]. Access to routinely collected data from emergent hospital EPR systems could solve this problem. Linkage of EPR data across primary and secondary care would enable examination of health problems managed in both settings. Indeed, NHS England’s care.data program plans to collate general practitioner records and link to hospital records on a national scale, significantly increasing the volume and depth of data for research and other uses [19]. In time, wider linkage to other information such as social care, dental records, and biobanks will progress [20]. This paradigm shift in “big data” would expand research opportunities, but, as the public response to care.data revealed [21,22], it also raised important challenges in terms of patient confidence and trust in how EPRs are used in medical research. These challenges include anonymity and the role of consent. When more and more parts of an individual’s information are pieced together, even if anonymized, the chances of reidentification increase [23]. As more datasets are linked and whole genome sequencing becomes part of standard clinical care, this problem will worsen [24], and risk loss of public trust.

Personal data are routinely collected in the NHS with patients’ implicit consent, with data processing governed by the Data Protection Act (DPA). Access to personal health care data is permitted only for those directly involved in their care. Informed, explicit, and voluntary (opt-in) consent is required for access to identifiable patient-level data for research. However, consent is not required when anonymized data are used for research. Linkage of personal data from primary and secondary care by the care.data program does not require patient consent under the Health and Social Care Act (2012). This individual-level data is only subject to limited anonymization [22]. Nevertheless, a fair processing obligation under the DPA requires that data subjects know what happens to their data. NHS England’s two main approaches to ensure fair processing are: (1) an opt-out process with the default assumption that routine NHS data can be used for approved research, and (2) a public awareness campaign to inform patients of data processing and use. There have been criticisms of both.

## Opt-Out Versus Opt-In

Opt-out makes the moral assumption that people are content for their anonymized health data to be used to benefit public health. However, anyone who objects to sharing data outside the NHS, or to sharing certain types of data, will have to opt-out of sharing any information with anyone [25]. Mass opt-out, perhaps worsened by misunderstanding of the risks, could result in a marked reduction of potential participants and threaten research validity. It is worth noting that opt-in systems also have challenges of uptake and representativeness for population research. Furthermore, proposed amendments to the European Union Data Protection Directive (95/46/EC) may render opt-out unlawful [26]. Opt-in relies on active patient participation. Some evidence shows that this is what people expect, despite not being legally required [15,27]. It avoids problems arising through patients feeling lack of control over the fate and flow of their electronic data [28].

## Knowledge of the Data Recipients

In early 2014, care.data ran a public awareness campaign including a national leaflet delivery, a patient telephone information line, and social media activities. These described how health data from primary and secondary care EPRs may be used, who might receive it, provided reassurance on the safeguards in place, and explained how to opt-out. The campaign received criticism for not adequately conveying its benefits and safeguards [29]. Although the campaign met the DPA fair processing requirements and Caldicott 2 review recommendations [30], the population-level approach lacked reassurance of individual patient data flow. Advocates believe studying deidentified data in safe havens does not threaten confidentiality, but the public understanding of data safe havens is questionable, and needs proper explanation. Access by “other approved organizations” remains a grey area, raising concerns for potential participants [31]. At the time of writing, care.data rollout had been deferred [32]. The observed discourse between patients’ general support for reuse of routine data for research and concerns raised around care.data may be explained by the ambiguous nature of the information disseminated in its awareness campaign. Also, the positive attitudes revealed from research cannot be easily translated to the care.data campaign; caution should be heeded in doing so, and experience warrants independent research.

## Dynamic Consent in Medical Research

A solution to the challenges described above is to move to a more effective, on-going model of patient information and consent [33]. Using consent as the basis for sharing medical data stored in EPRs addresses the technological limitations associated with anonymization techniques, while also respecting the autonomy of patients. Making it dynamic could allow patients to more readily provide or withdraw their consent over time, while also providing information to patients about how their personal data are used. This could list data recipients, and demonstrate how EPR data sharing has contributed toward better health care by providing lay summaries of research results from the studies toward which their data have contributed.

**Figure 1:**
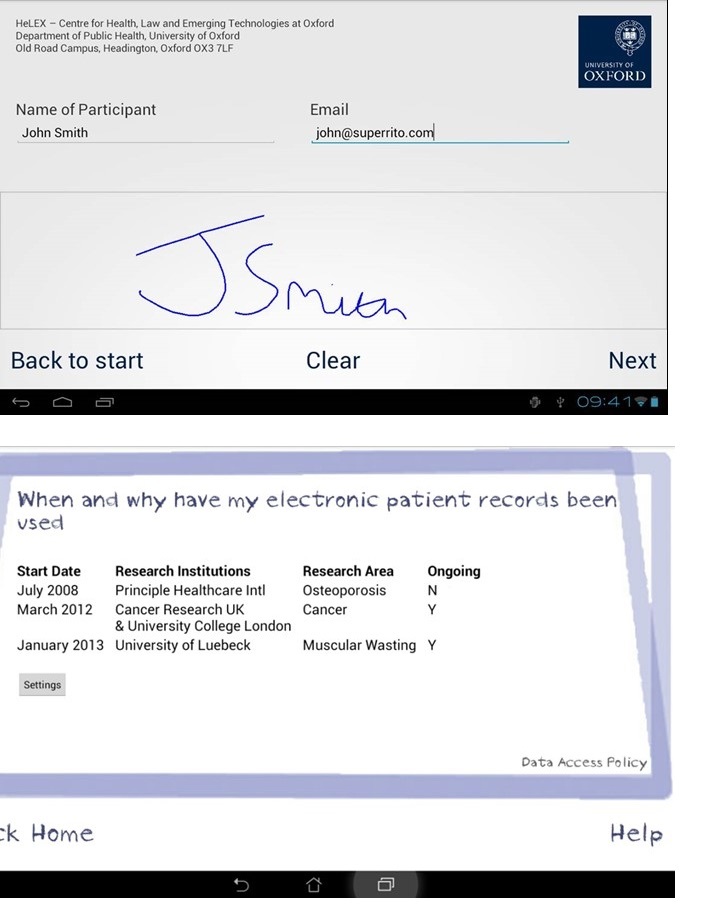
Dynamic consent interface. The origin of the image is from HW Communications and the University of Oxford.

The Dynamic Consent model [34] is one solution that provides a participant centered approach to consent (Figure 1). It provides additional functionality by exploiting technology to allow on-going engagement and maintenance of research participants’ consent preferences (“expressions”). The UK Ensuring Consent and Revocation project [35] developed a prototype comprising privacy-enhancing technologies, such as policy-driven privacy-aware access control and obligation management, within an overall Web 2.0 compatible technical architecture. State of the art enterprise information technology and cryptographic techniques wrap and bind patient information with consent expressions and enterprise policies. Novel techniques such as attribute-based encryption [36], identity-based encryption [37], and functional encryption [38,39] each provide suitable techniques for the binding of different classes of metadata with information, and the control of access to that information. Information can flow throughout and between health care information systems, while assuring patient information is handled in accordance with regulations. Patients could also track and audit their information usage, change privacy settings, and choose how and when they are contacted [34]. It enhances confidence by passing control to patients, with data flow controlled at the level of consent, thereby avoiding the broad opt-out, which does not satisfy a patient’s wish to allow access to some groups/projects, but not others (ie, academic researchers, but not the pharmaceutical industry). It also avoids problems arising through patients feeling they have no control over the fate of their EPR, and because of this, may avoid seeking treatment through fear of insurance refusal, loss of employment, or stigmatization [28]. The implementation of Dynamic Consent through a convenient computer-based interface allows for the possibility of using videos, animation, and other formats to increase the communication to the patients, including the presentation of lay summaries of research results.

A legitimate concern raised by Dynamic Consent is that it may present new ethical questions around user coresponsibility and social exclusion [13]. Representative uptake might be problematic, as groups with lower socioeconomic status may be less likely to engage with opt-in models [21,22,40].

At the institutional level, Dynamic Consent implies an e-infrastructure that is able to collect consent, to allow data permissions to direct the flow of data to recipients, to capture a complete audit trail of data recipients, and to receive comprehensive, up-to-date lay summaries of research findings to feed back to patients. Scalability is thus constrained by the provision, maintenance of such systems, and infrastructure. We are currently exploring hospital patients’ perceptions of health information security, and the role of consent in health data use and EPR access, with positive early results.

## Conclusions

Dynamic Consent alone will not adequately provide a population of well informed, engaged, and e-Health literate research participants. However, it does provide a platform to develop the ethical and engagement framework to ensure respect for the rights, needs, and expectations of diverse participants. It may also play a role in widening participation in an age where health care is increasingly characterized by digital innovations. As the experience of care.data has shown, public trust is fundamental to the successful use of data held in EPRs. Dynamic Consent may provide a transparent, flexible, and user-friendly means to inform and maintain that trust.

## Abbreviations

CPRD: Clinical Practice Research Datalink
DPA: Data Protection Act
EPR: electronic patient record
NHS: National Health Service
